# Reconstruction of deep and perforating corneal defects in dogs—A review (Part I/III): Autogenous ocular tissues, donor tissues, and corneal clarity scoring

**DOI:** 10.1111/vop.13286

**Published:** 2024-10-01

**Authors:** Rick F. Sanchez, Eric C. Ledbetter, Marta Leiva

**Affiliations:** ^1^ EBVS® Specialist, Dipl ECVO, DVM, CertVetEd/FHEA, Specialistische Dierenkliniek Utrecth (SDU‐AniCura) Utrecht The Netherlands; ^2^ DVM, DACVO, Department of Clinical Sciences, College of Veterinary Medicine Cornell University Ithaca New York USA; ^3^ EBVS®Specialist, Dipl ECVO, PhD, DVM, Servei d'Oftalmologia, Hospital Clínic Veterinari Campus Universitat Autònoma de Barcelona Barcelona Spain; ^4^ Departament de Medicina i Cirurgia Animal, Facultat de Veterinària Universitat Autònoma de Barcelona Barcelona Spain

**Keywords:** conjunctival pedicle graft, corneal clarity score, corneoconjunctival transposition, corneolimboconjunctival, transposition, non‐penetrating keratoplasty, penetrating keratoplasty

## Abstract

Corneal reconstruction is a key part of veterinary ophthalmic practice and numerous reconstructive techniques have been described for use in small animals in the peer‐reviewed veterinary literature written in English. Despite the evidence accrued over the last six decades in over 40 clinical articles and numerous other publications on ocular surface health, several key areas require further study. The comparison between studies is difficult due to elements that go beyond common factors, such as the indication for surgery, the reconstructive technique preferred by the surgeon or the availability of reconstructive materials. However, the differences in reporting style adopted by different authors between similar studies and the lack of data found in retrospective studies add to this complexity. The present review is divided into three parts. One covers the use of autologous materials for reconstruction and corneal transplants, as well as corneal clarity. A second part focuses on biomaterials and keratoprosthetics, while the third part focuses on the use of corneal sutures and report of ocular discomfort/pain in the veterinary literature. The review focuses on the main findings of each reconstruction technique. It aims to identify areas where key information about common procedures is missing so that general guidelines may be provided for the planning of patient record keeping and future retrospective or prospective studies, while it also aims to highlight the presence of knowledge gaps that deserve further attention.

## 1 INTRODUCTION

Ulcerative keratitis or corneal ulcerative disease (CUD) in dogs is a potentially devastating corneal condition that causes pain and affects vision and the patient's quality of life.^1^ This encompasses a wide range of clinical presentations and may be divided into superficial and deep ulceration, descemetoceles, and perforations that include leaking descemetoceles.^1^ Collagenolysis is a complication of CUD and may be localized to an area of the cornea or be widespread.^1^

A study in the United Kingdom that included the general practice medical records of over 104 000 dogs spread over 110 clinics reported a 1‐year prevalence of CUD of 0.8%, with purebred dogs having 2.23 times the odds of developing CUD compared to non‐purebred dogs.^2^ Moreover, it confirmed the long‐held suspicion that certain breeds appeared to have a predisposition to CUD, with brachycephalics and spaniel types having, respectively, 11.18 and 3.13 times the odds of developing CUD compared to non‐purebred dogs, while the Pug, Boxer, Shih Tzu, Cavalier King Charles Spaniel, and Bulldog had the highest prevalence (i.e., in descending order).^2^ The complex set of risks associated with brachycephaly that predispose to CUD is known as brachycephalic ophthalmic syndrome (BOS), and it has been thoroughly reviewed elsewhere.^3^ Dogs may also develop CUD in association with entropion,^4^, ^5^ distichiasis,^6^ ectopic cilium,^7^, ^8^ dry eye disease,^9^, ^10^ corneal degeneration,^11^ trauma,^12^, ^13^ and ocular surface overexposure secondary to a variety of problems.^14^, ^15^, ^16^, ^17^

Deterioration of CUD through bacterial colonization and corneal inflammation may lead to corneal melting (i.e., collagenolysis or keratomalacia), deepening of the ulcer bed and corneal perforation, complications that are likely in dogs with more than one risk factor, such as BOS and dry eye disease.^3^, ^10^ While primary forms of corneal ulceration also exist,^18^, ^19^, ^20^, ^21^ these rarely develop into deep corneal ulceration unless they succumb to bacterial colonization and/or collagenolysis.

Surgical reconstruction of corneal ulceration is strongly recommended when the ulcer bed has reached 50% of stromal depth.^1^ Despite the existing general knowledge on CUD in dogs, a study found that only 7.4% (62/834) of affected dogs were referred for further assessment and that there was no difference in referral rates for brachycephalic versus non‐brachycephalic dogs, or spaniel versus non‐spaniel types.^2^ While surgery was more likely to be implemented in cases that were referred, the study also found that resolution was less likely when referral was sought, with one of the possible explanations being the late recognition of urgent and deteriorating CUD.^2^ It would seem sensible for specialists to encourage early referral of these patients and even consider reconstructive surgery in cases of CUD with multiple risk factors. To guide advice, specialists need to have a thorough, evidence‐based understanding of corneal reconstructive options and their expected outcomes. Specialists must compare and contrast the findings between published studies, but notable differences in the reporting style and the intrinsic limitations of study design make this task rather challenging.

Several techniques for corneal reconstruction have been developed for use in dogs and may be categorized by the material used in the reconstruction, such as autogenous ocular tissues, homologous and heterologous donor tissue, and what may be grouped under the broad category of biomaterials. Lastly, is the less well‐known category of keratoprosthetics. Each is covered separately in this review, which has been divided in three parts (i.e., autogenous ocular tissue, corneal donor tissue, and clarity scoring in part I, biomaterials and keratoprosthetics in part II, and the reported use of microsutures and ocular discomfort in the veterinary literature in part III). The use of evidence‐based observations to discuss what might be a “superior” or most adequate method of corneal repair poses many challenges, as choice of surgery often depends on factors that are independent of the needs of the patient, such as the availability of the material to carry out the repair, the costs involved, and surgeon's preference. Comparing outcomes is also complex, as definitions and other parameters vary between studies (e.g., ulcer depth, what constitutes success and failure, differences in follow‐up times, and corneal clarity scoring). Despite these differences unifying trends also exist, with early reports focusing on saving the globe,^22^ and later reports stressing the importance of corneal clarity (i.e., preserving vision).^23^, ^24^, ^25^

The present review aims to report the findings and outcomes of the articles published in each category, and to identify the potential challenges that specialists find when writing about corneal reconstruction so that these may be minimized in future studies. The review also aims to identify knowledge gaps and encourage further studies on these areas.

A search of the peer‐reviewed veterinary literature written in English in the last 61 years (i.e., start of 1962 to the end of 2023) and that focused on corneal reconstruction for spontaneous CUD of various causes in canine patients was carried out to find out how many articles existed. The author employed a commonly used a search engine (i.e., PubMed) and included terms such as ‘corneal ulcerative disease’, ‘corneal reconstruction’, ‘corneal surgery’, ‘corneal graft’, ‘conjunctival pedicle flap’ (and graft), ‘conjunctival patch graft’, ‘corneolimboconjunctival transposition’, ‘corneoconjunctival transposition’, ‘third eyelid graft’, ‘buccal mucosal graft’, ‘biomaterial’, ‘amnion’, ‘renal capsule’, ‘bovine pericardium’, ‘porcine intestinal submucosa’, ‘Biosist’, ‘porcine urinary bladder’, ‘keratoplasty’(lamellar and penetrating), ‘corneal melting’ and/or ‘keratoprosthesis’, combined with the terms ‘dog’, or ‘canine’. Cross referencing was also carried out.

## 2 AUTOGENOUS OCULAR TISSUES

The use of the animal's own tissues for corneal reconstruction has several obvious advantages including tissue availability without the need to have a donor bank or the costs typically associated with the purchase of commercially available biomaterials, and the lack of an immune response against the tissue used.^22^, ^24^, ^25^, ^26^ However, depending on the surgical technique chosen, there might be tissue availability limitations and the donor site will sustain a certain degree of surgical trauma. Autogenous tissues typically include bulbar conjunctiva for conjunctival pedicle flaps and conjunctival island grafts, palpebral tarsoconjunctiva for pedicle flaps and island grafts, third eyelid cartilage‐conjunctiva for island grafts, corneal buttons of corneal epithelium and superficial stroma for nonpenetrating keratectomy grafts, and cornea with limbus and conjunctiva or sclera for corneoscleral and corneo‐limbo‐conjunctival transpositions (CLCTs).

A total of 14 studies including 760 canine eyes are included in the peer‐reviewed veterinary literature written in English on this topic. The success rates range from 83% to 100%, with the majority of the studies reporting success in over 90% of cases.

### 2.1 Bulbar and third eyelid conjunctiva

Kuhns 1979 described the use of conjunctival “patch” (i.e., “island”) grafts in 10 canine eyes, 9 of which were of brachycephalics (i.e., all Pekingese coincidentally).^26^ Hakanson and Meredith 1979 described the use of conjunctival pedicle “grafts” in 27 eyes, 18 of which (66.66%) were of brachycephalic breeds and 2/27 (7.40%) that were characterized as a spaniel, bringing the total number of affected eyes of brachycephalic and spaniel types to 20/27 (74%).^22^ Hakanson et al 1988 followed this up with further comments on the use of conjunctival pedicle “grafts” in 69 additional eyes, 51 of which (73.91%) were reported to be in “exophthalmic” breeds.^27^ Lastly, Dorbandt et al compared the outcome of conjunctival pedicle flaps on their own (37 eyes) to conjunctival pedicle flaps that also used porcine biomaterial (36 eyes).^28^ The authors included a total of 44/73 (60.27%) eyes of brachycephalic dogs and 4/73 (5.47%) eyes of spaniel breed dogs, bringing the total number of affected eyes of brachycephalics and spaniel types together in that study to 48/77 (65.75%).^28^ Articles that reported using a conjunctival flap as support to the main graft, such as with third eyelid cartilage‐conjunctival grafts,^29^ with porcine biomaterial^30^ and with autologous buccal mucous membrane grafts^31^ will be reviewed in the biomaterial section of this article.

The bulbar conjunctiva was the graft donor site in all the cases of the conjunctival patch grafts reported by Kuhns in 1979, while the third eyelid conjunctiva was used in a single case that required a second graft that overlapped the first.^26^ The study included four perforations (4/10 [40%]), and the rest of the cases (6/10, 60%) included three deep ulcers, two descemetoceles, and one ulcer that was not specified but was presumed to be deep or a descemetocele.^26^ Microbiologic assessment was not reported for any of the cases and success, which was defined as “preservation of the anterior chamber and functional vision,” was reported at 90% (9/10), with graft failure in one case and part failure in another case that healed without further problems.^26^ The authors included different follow‐ups between several weeks to several months and images of the eyes at last revaluation. All the cases appeared to have central to paracentral ulcers, and all but one appeared to have an opaque cornea at the site of the graft. The authors described nearly all the eyes as either having vision, being partly visual or having at least peripheral vision.^26^

The conjunctival pedicle “grafts” reported by Hakanson and Meredith in 1987^22^ and Hakanson et al in 1988^27^ described success as “maintaining ocular integrity,” “maintaining graft integrity” and as having “limited corneal opacification,”^22^ and as preservation of ocular integrity and having no graft dehiscence.^27^ There were a total of 25/27 (92.59%) ulcers in the earlier study that affected the central (20/27,70.07%) or paracentral (5/27, 18.51%) cornea, including 15/27 (55.55%) eyes presenting with descemetocele and 4/27 (14.81%) eyes presenting with aqueous leakage.^22^ The authors of the second study did not mention what part of the cornea was affected, though they reported a total of 18/74 (24/32%) perforated ulcers and at least 15/74 (%) descemetoceles (i.e., the authors did not separate between the 69 dogs and 5 cats included).^27^ Microbiologic assessment was not reported in either study. The success rates, which did not separate between dogs and cats in either study, were similar, at 32/35 (91%)^22^ and 71/74 (96%),^27^ respectively. The authors of the first study reported that in 25/32 (78%) eyes in which there was a follow‐up there was “a clear or much cleared cornea” around the graft, and that a scar was expected at the site of the graft given the nature of the surgery.^22^ They also reported that perforations had a higher chance of failure and that graft direction of more than 45 degrees from vertical was identified as a risk for failure.^22^ The authors of the second study added that leakage in perforations was associated with the loss of single sutures and that excessive stretching between opposing sutures appeared to increase the risk for failure.^27^ Lastly, it is worth noting that although the technique was reported as a “graft,” a source of blood supply was preserved through a tissue bridge still connected to the original donor location, which qualifies it as a “flap” (i.e., conjunctival pedicle flap).

The study by Dorbandt et al compared conjunctival pedicle flaps alone (*n* = 37) to conjunctival pedicle flaps in combination with porcine biomaterial (*n* = 36) and included 36/73 (49.31%) perforations, 19/73 (26.02%) descemetoceles and 17/73 (23.28%) deep ulcers in addition to 1 case with an unspecified depth (1/73, 1.37%).^28^ The authors reported that the lesion affected the central cornea in more than half of the cases, that the overall success rate was 68/73 (93%) and that there was no difference in outcome between the two groups (i.e., pedicle flaps alone or with biomaterial), whether or not there was a perforation.^28^

### 2.2 Palpebral tarsoconjunctiva and third eyelid cartilage‐conjunctiva

The use of autogenous tarsoconjunctival pedicle grafts was described by Peiffer in 1975 in two poodles, one of them with dry eye.^32^ Autogenous tarsoconjunctival island graft were described by Scagliotti in 1988 and included 37 eyes but the breakdown by breed was not included.^33^ Lastly, the use of a combination of an autogenous graft of nictitating membrane cartilage and conjunctiva that also had an overlying conjunctival pedicle graft was described by Blogg et al in 1989 to repair full and partial‐thickness corneoscleral surgical wounds created during limbal melanoma excision in four eyes of four German Shepherd dogs.^29^

The autogenous tarsoconjunctival grafts described by Peiffer utilized the upper eyelid to treat deep central or paracentral corneal ulcers in two dogs and the grafts were transected after 3 weeks, when the grafts were reported to have been incorporated into the recipient cornea.^32^ Success was not defined but the purpose of the grafts was described as “preserving ocular integrity,” “minimizing irreversible disease of the cornea,” and “not interfering with vision.”^32^ Microbiologic assessment was not reported for either case, but the eyes healed and were reported to be visual despite the expected corneal scar, which had shrunken 4 weeks after transection in one of the cases.^32^

The autogenous tarsoconjunctival grafts described by Scagliotti also utilized the upper eyelid in 37 canine eyes, and the authors argued that unlike the tarsoconjunctival pedicles, island grafts did not require a second procedure to transect the pedicle.^33^ The cases included 8/37 (21.62%) deep ulcers, 15/37 (40.54%) descemetoceles, and 14/37 (37.83%) perforations, with 10/14 (71.42%) of the perforations being centrally located.^33^ The results did not include microbiologic assessment, but included seven feline eyes and the authors described graft acceptance occurred in 43/44 (97.72%) of all the eyes.^33^ While the author expected to have an obvious scar given the nature of the graft, partial vision was restored in 40% of the eyes with perforated corneas that were not visual before surgery.^33^

Lastly, the grafts described by Blogg et al. utilized a pedicle (*n* = 2) or an island graft (*n* = 2) that were harvested from the bulbar aspect of the nictitating membrane and were sutured into corneoscleral defects created after limbal melanocytoma removal in four dogs.^29^ All of the eyes maintained vision with follow‐up times ranging from 1 month to 26 months, and while some had relatively large lesions, they did not affect the central cornea extensively.^29^

### 2.3 Corneal transpositions

Transpositions typically relocate tissues from a healthy donor location to the recipient site, where a defect is located, in the same eye.^24^, ^25^, ^34^, ^35^, ^36^, ^37^, ^38^, ^39^ Theoretically speaking, surgeons could consider a donor site from the fellow eye when performing an island graft if this proved to be advantageous. A number of different transpositions have been described in the veterinary literature for use in dogs. These include the relocation of corneal stroma with overlying epithelium,^34^, ^35^ of corneo‐limbo‐sclera,^36^ or of corneo‐limbo‐conjunctival tissues.^24^, ^25^, ^37^, ^38^, ^39^ The use of CLCTs with porcine biomaterial (ACell®) has also been reported in one study.^40^

Brachycephalic breeds were overrepresented in most studies with corneal transpositions that offered information on patient signalment. The two largest studies included in this section reported that brachycephalics were affected in 65% (65/100 cases)^39^ to 77.75% (325/418 eyes).^24^ Similar or higher percentages were described in reports with a lower number of patients.^25^, ^35^, ^37^, ^40^

Lamellar corneoscleral transpositions were described by Parshall in 1973 in six eyes of five dogs, and the author defined success as maintaining the eye and preserving visual function and reported anatomical success in 5/6 (83.3%) of the eyes, though all but one had some degree of corneal scarring at the surgical site.^36^

A modification of this technique that included the cornea, limbus, and conjunctiva (i.e., all in one graft without incorporating sections of the sclera) was described in the “Veterinary Ophthalmology Notes” from Doctor Severin in 1976.^38^ This was known as corneoconjunctival transposition or CCT. The largest study on this technique to date was published in 2021 and recommended the use of the term corneolimboconjunctival transposition or CLCT.^24^ The authors argued the limbus is a unique histological structure of the ocular surface, that it requires specific attention for its dissection during surgery, and that it remains visible externally as a white or black line after healing.^24^ This section of the review uses the term CLCT to refer to cases reported as CCTs or CLCTs.

A conference abstract in 2007 reported the use of CLCTs in three eyes of brachycephalic dogs with keratoconjunctivitis sicca, and a good outcome was reported in all three cases except for the development of pigmentation in a Pug.^37^ Then, the first peer‐reviewed, retrospective study was published in 2020 including 16/100 (16%) deep ulcers, 33/100 (33%) descemetoceles, and 51/100 (51%) perforations.^39^ It described preservation of vision and clarity as its goals and reported that the technique had been effective in all corneal wound types, and that vision was preserved in 96/100 (96%) of patients after a median follow‐up time of 188 days.^39^

The largest CLCT study to date included 418 eyes in 399 dogs with deep (317/418, 75.83%) or perforated corneal ulcers (101/418, 24.16%), and 122/418 eyes (29.19%) that had been diagnosed with keratoconjunctivitis sicca.^24^ It reported success as a presence of a menace response, lack of glaucoma, or endophthalmitis in 406/418 (97.13%) eyes after a mean follow‐up time of 100 days.^24^ The study also found that pre‐existing perforation significantly increased failure, something that had not been noted before, possibly due to the lower case numbers in previous reports.^24^

The study that combined CLCTs with porcine biomaterial included a total of 19 eyes (18 dogs) with 7/19 (36.84%) deep ulcers and 12/19 (63.16%) perforations.^40^ It was the first CLCT study to report culture and sensitivity results of all the cases.^40^ The authors described there was positive growth for 8/19 (42.10%) of the dogs tested and that *Staphylococcus intermedius* was found in 4/19 (21.05%) dogs, *Streptococcus canis* in 2/19 (10.52%) dogs, and that one dog each (1/19, 5.26%) showed *Klebsiella pneumoniae*, *Pseudomonas aeruginosa*, or *Staphylococcus schleiferi*, while one dog had both *Streptococcus canis* and *Pseudomonas aeruginosa*.^40^ Keratoconjunctivitis sicca was present in a large proportion of these cases (10/19 eyes, 52.6%).^40^ There was a median follow‐up time of 188 days, and integration of the transposition was observed at a median of 20 days (range 7–38 days) with no statistically significant difference between the comparison groups.^40^ The authors reported that a positive menace response was present in the last re‐evaluation in 17/19 (89.47%) eyes with the two patients left out having developed diabetic cataracts in one case and cataracts suspected to be linked to the history of corneal perforation in the other case.^40^

A case series in 2023 introduced the use of multidirectional CLCTs in seven dogs (i.e., seven eyes).^25^ Bacteriology had been carried out in all the affected eyes and showed no growth in 2/7 (28.57%) dogs while the rest had *Pseudomonas aeruginosa* (3/7, 42.86%) or *Staphyloccoccus* spp. (2/7, 28.57%).^25^ The study included a follow‐up of at least 60 days after which all the animals were reported to be sighted.^25^

There were only two studies that reported the use of autogenous anterior lamellar corneal grafting in dogs. One included 16 experimental anterior lamellar grafts to repair full‐thickness defects in 9 healthy dogs, and 7 anterior lamellar grafts to repair deep, nonpenetrating, anterior corneal lesions in one eye of 7 patient dogs.^34^ The study defined success as the ability to visualize the retina through the graft once the eye had healed, and reported that all eyes healed well and that there was a successful outcome in all the patients except one that had progressive pigmentation.^34^ The authors of this study performed electron microscopy 3 weeks after surgery in two of the healthy eyes that were experimentally grafted and showed that endothelial cells had grown under the grafts and that they had cellular changes consistent with polymorphism and polymegathism, suggesting migration from the surrounding cornea onto the underside of the grafts.^34^ The other study included 16 eyes and reported a successful outcome in 14/16 (88%).^35^ Bacteriology had been carried out in 8/15 (53.33%) of the cases and showed that 3/8 (37.5%) had no growth, that 3/8 (37.5%) cases had *Stapyloccoccus pseudointermedius* and that one dog each (1/8, 12.5%) had either Group G *Streptoccoccus* or *B‐hemolytic streptococcus*.^35^

The maintenance of graft clarity after surgery is a pivotal aspect of transpositions. It is noteworthy that postoperative pigmentation was reported in several of the transposition reports.^24^, ^25^, ^34^, ^35^, ^37^, ^39^, ^40^ One of the cases in the Brightman et al study had a pigmented cornea prior to surgery and developed pigment over the graft during the healing process, as the authors had anticipated.^34^ Euryblepharon and pigmentary keratitis were significantly associated with an increase in graft opacification in one study,^39^ while corneal clarity scoring was lower for Pugs compared to the rest of the dogs in another study.^24^

Lastly, epithelial inclusion cysts were reported in three of the transposition studies, with two of the smaller case series reporting a cyst in 5/14 (35.71%)^40^ and 2/12 (16.66%)^25^ cases, and the largest of the case series reporting a cyst in 22/418 (5.3%) of the eyes treated.^24^

## 3 DONOR TISSUE

### 3.1 Homologous and heterologous donor corneas

Dr. Bigger was the first ophthalmologist to describe, in 1837, corneal grafting in veterinary medicine,^41^ but it was not until 1957 that Dr. Holt performed the first documented homologous corneal graft in a dog.^42^ Since then, corneal grafting surgery and postoperative care have evolved dramatically, making keratoplasty a valuable option for restoration of corneal integrity and vision in many ocular diseases. Despite this, the published clinical studies on the use of keratoplasties for deep or perforating corneal conditions in dogs are scarce,^34^, ^35^, ^43^, ^44^, ^45^, ^46^, ^47^ suggesting that corneal grafting is not yet widely used in canine patients. This fact could be associated with some the challenges associated with keratoplasty. A pivotal challenge includes the identification of donors that meet the selection criteria, the appropriate harvesting of the donor eyes and successful preservation of the corneal tissue. Another significant challenge is associated with the intricacies of the surgical procedure. Last but not least, there is a lack of a standardized postoperative protocol for prevention and treatment of corneal rejection in the dog.

#### 3.1.1 Identification of donors, harvesting and preservation of corneal tissue

Keratoplasty is typically considered to have superior outcomes and lower requirement for immunosuppression compared to “solid organ” transplants because of the inherent immune privilege and tolerogenic mechanisms associated with the anterior segment of the eye.^48^ *Corneal transplantation* guidelines in humans do *not* support *corneal matching* due to the extensive immune privilege of the *cornea*.^48^ More recently, sex‐mismatched recipients have been associated with a significantly high risk for rejection than sex‐matched recipients, which suggests the possibility to additional rejection mechanisms.^49^

Similarly, the immune privilege of the anterior segment greatly simplifies the donor identification in dogs. However, obtaining canine corneas is still challenging as tissue availability is dependent on a well‐informed community of pet owners that will consent to donation. In addition, the conditions for being a donor are restrictive. Ideally, donors should be young or middle‐aged dogs that died for other reasons than neoplastic or infectious systemic diseases and had no evidence of ocular disease.^45^, ^46^, ^50^, ^51^ Unfortunately, the lack of an international canine eye banking system hinders the acquisition of corneal tissue that may be used for emergencies. Some institutions have developed in‐house eye banks to meet the needs of their canine patients.^45^, ^46^, ^47^, ^50^, ^51^, ^52^ These banks attempt to lengthen the storage time of corneal tissue to increase the pool of available corneas tissue by focusing on the preservation of corneal integrity and viability, but often rely on xenografts (i.e., grafts from other animal species). Some also opt to preserve the entire globe instead of the corneal button, thus providing scleral tissue as well.^46^, ^50^, ^51^

Based on origin, keratoplasties may be classified into homologous corneal grafts (HoCG), homografts, or allografts, when the donor tissue comes from an animal of the same species as the recipient, heterologous corneal grafts (HeCG), heterografts, or xenografts, when the tissue comes from an animal of a different species, and autologous corneal grafts (ACG) or autografts, when the donor and recipient are the same animal. These three types of keratoplasties have been used in dogs with deep and perforating corneal defects with successful results in terms of globe and vision preservation.^34^, ^35^, ^43^, ^44^, ^45^, ^46^, ^47^ Autologous corneal grafts are also discussed in the “Corneal transpositions” part of the “Autogenous ocular tissue” section of this review.

Based on the preservation method used and the duration up to which the corneal tissue can be viably stored, donor tissues may be classified as fresh, including “*short‐term”* (i.e., up to 48 h), “*intermediate‐term”* (i.e., 2–4 days), and “*long‐term”* (i.e., average of 25 days), and frozen, including “*very long‐term”* (i.e., years). Studies that focus on storage methods of canine corneas are scarce.^51^, ^53^, ^54^, ^55^, ^56^ Some additional information may be extrapolated from clinical retrospective studies that suggest the moist‐chamber storage at 4°C is the most commonly used method for canine “*short‐term”* corneal preservation using the entire eyeball or a corneal button.^44^, ^45^, ^46^, ^47^, ^50^ In addition, conservation in Optisol® GS medium at 4°C has been shown to successfully preserve the corneal endothelium from 10 to 21 days.^55^, ^56^ “*Long‐term”* preservation methods refer to organ cultures that maintain endothelial cell function of human corneas stored at 34°C for at least 5 weeks.^57^ Unfortunately, most veterinary practices do not have the required equipment to guarantee the proper maintenance of biological cultures; thus, “*very long‐term”* preservation methods are most frequently preferred. Although not ideal, “*very long‐term”* preservation methods are currently the most used by veterinary ophthalmologists, accounting for up to 90% of keratoplasty surgeries in dogs.^46^ Cryopreservation through moist‐chamber storage with a broad‐spectrum antibiotic solution at freezing temperatures (i.e., −20°C to −30°C) have been successfully used for homografts^44^, ^45^ and porcine heterografts.^46^, ^47^, ^50^ The main limitation of corneal cryopreservation is the death of endothelial and epithelial cells, limiting preserved tissue to corneal stroma used for tectonic support. However, cryopreservation could potentially extend the use of the donor tissue for 8 years without structural or microbiological impediment.^51^ More recently, dehydrated heterografts have been successfully used in penetrating keratoplasties in dogs, showing no statistically significant differences with cryopreserved corneal tissue.^47^ Thus, dehydrated cornea could be considered an alternative to cryopreserved grafts for the management of full‐thickness corneal defects.

Last but not least, autografts have been successfully used for the treatment of deep corneal defects and perforations in the dog in selected patients who possess enough healthy cornea tissue to be transposed onto the ulcer bed as a donor graft.^34^, ^35^ Interestingly, corneal endothelium was shown to migrate and cover the intraocular side of the autograft in one study that used electron microscopy.^34^ However, our knowledge of how effective this mechanism might be, and what variations there may be between dogs of different ages and/or breeds remains largely unknown. Autografts offer potential advantages over conventional homografts/heterografts, in particular the tissue availability and the lack of the risk of immunological rejection. Conversely, the additional surgical injury induced onto an already affected cornea has the potential to worsen the original corneal problem.

### 3.2 Intraoperative considerations and particularities of keratoplasties

The two types of keratoplasties reported for reconstruction of deep and perforating corneal defects in dogs are anterior lamellar keratoplasty (ALK) and penetrating keratoplasty (PK), respectively. The ALK technique includes superficial and deep lamellar keratoplasties (i.e., SALK and DALK). While corneal stroma is preserved for use in ALK (i.e., no endothelium is grafted), the stroma and endothelium are preserved in PK to help recover corneal transparency. As a general rule, ALK with fresh allograft or frozen allograft or xenograft is recommended to treat deep corneal ulcers with recipient endothelium that is still viable. Conversely, fresh allograft is preferred in corneal perforations when maintenance of transparency is a goal of the surgery, as it provides viable endothelium. Nevertheless, the rejection rate of fresh allografts is higher and must be managed accordingly. A frozen allograft or xenograft may be recommended for tectonic support of PKs when no fresh tissue is available.

Specific keratoplasty instrumentation is recommended for ALK and PK procedures in addition to the standard corneal instruments. A keratoplasty set should include an eyeball stand (i.e., if the entire globe was preserved), a corneal cutting Teflon block, corneal trephines of different sizes (i.e., for donor and recipient tissue, with or without pre‐set depth), and a suture placement marker and different blades (i.e., crescent knifes or similar, with/without pre‐set depth).^58^, ^59^ The Flieringa scleral fixation ring that is commonly used in the recipient eyes of human patients^60^ is not necessary in dogs because it does not prevent the collapse of the canine anterior chamber due to anatomical differences.

The preparation of the donor graft is generally performed prior to the preparation of the recipient cornea, in order to reduce the exposure time of the intraocular contents. Nevertheless, in some deep corneal ulcers, the preparation of the corneal bed as the first surgical step is preferred to allow for a better delimitation of the wound margins, and therefore a more accurate selection of the size of the donor graft.^45^, ^46^ In order to avoid complications associated to postoperative graft retraction, 0.5‐1 mm oversized donor button is recommended for both ALK and PK.^46^ In ALK, the thickness of the cryopreserved graft should be slightly less (70–80%) than that of the recipient cornea, anticipating the postoperative graft edema.^46^ In addition, the removal of Descemet's membrane from the donor cornea is recommended because attempts to implant Descemet's membrane in humans have shown poor healing between the recipient bed and the donor button. Thus, harvesting deep lamellar buttons is not recommended for lamellar ALK.^61^

Preparation of the donor graft in cryopreserved corneas (ALK and PK) is usually performed through an *ab externo* surgical approach, while if fresh grafts are used for PK, a standard *ab interno* approach is preferred in order to improve the endothelial sectility.^46^

Absorbable and non‐absorbable monofilament suture materials have been used in canine keratoplasties. In general, tissue reactivity tends to be lower with non‐absorbable sutures, which reduces corneal neovascularization and therefore the rejection rate of tissue that is foreign to the host.^44^ The main drawback of non‐absorbable suture material is the need for suture removal, which requires sedation or general anesthesia in some dogs. While the surgeon can choose between simple continuous or interrupted suture patterns, the latter are the most commonly used in dogs with deep and perforated corneal ulcer, as they are considered to have a high risk of early suture loosening because of infection and inflammation.^44^, ^45^, ^46^, ^47^ The main disadvantage of the continuous suture technique is the potential for corneal graft wound dehiscence with suture failure, especially when a single continuous suture has been used. Burying knots has been previously suggested for human patients to reduce postoperative discomfort and neovascularization, and the knots may be positioned in the host or in the donor tissue, while avoiding the graft‐host interface.^62^ There is no consensus on the best direction to place the tails of the buried knots to decrease corneal neovascularization in grafting procedures in human patients.^62^ The third part of the present review focuses on the use of corneal sutures in small animal corneal reconstructive surgery including the reported use of the use of buried corneal sutures.

The postoperative use of a third eyelid flap as a tissue bandage for 15 days has been reported after keratoplasty in dogs.^45^ Imcharoon et al reported that corneal healing does not decrease when a third eyelid flap is used.^63^ One of the authors (ML) has observed that the “bandage” effect of the third eyelid on the corneal surface could decrease the postoperative corneal edema of cryopreserved grafts (unpublished data), something that warrants further study.

### 3.3 Postoperative protocol for preventing and treating corneal rejection

There is no standardization of the postoperative treatment, or of the rescue medication used in case of rejection, in dogs. This is mainly based on the fact that the rejection risk greatly differs among individuals, depending on parameters such as facial conformation (i.e., brachycephalic vs. non‐brachycephalic), age, corneal vascularization, corneal infection, concomitant ocular or systemic diseases, and type of graft (origin, size, location, preservation method, and thickness). The immune response may involve any or all the donor corneal layers. According to the literature, corneal cellular immunogenicity is mostly triggered by corneal endothelium, epithelium and to a lesser extent to the stroma.^64^ Changes brought about by the immune response to the epithelium or stroma are usually reversible, while the immune response to the endothelium usually leads to irreversible endothelial cell loss which often becomes the cause of graft rejection.^64^ This justifies the use of postoperative immunosuppression, especially in cases of fresh PK.

Postoperative treatment regimens differ greatly among reported studies, ranging from lack of immunosuppression,^45^, ^47^ to a simple‐therapy,^44^ a double‐therapy,^50^ or even triple‐*therapy immunosuppression*, including a calcineurin inhibitor, a cell cycle inhibitor, and corticosteroids.^46^ Publications on human patients suggest that immunosuppressive mono‐treatment is not enough for preventing host immune reaction against the graft material.^65^, ^66^ Cyclosporine‐A (CsA) is a calcineurin inhibitor that has immunosuppressive properties and may be used in the prophylaxis of transplant rejection. The combination of topical CsA and a corticosteroid in humans that have undergone PK does not prevent occurrence of graft rejection, but it reduces its incidence, especially in high‐risk keratoplasties.^65^, ^66^ A topical immunosuppressive treatment with a combination of a topical corticosteroid and tacrolimus has been successfully used in dogs.^50^ In addition, systemic administration of mycophenolate mofetil has shown similar^67^ to slightly better effectiveness^68^ than systemic CsA in preventing acute rejection following high‐risk corneal transplantation in humans, while having fewer side effects. A 15 mg/kg daily dose of mycophenolate mofetil was recommended for dogs in one study to reduce the potential toxic effects of the drug that might lead to gastrointestinal disease when given at higher end of the recommended doses.^46^ All in all, clinical evidence suggests the use of multiple therapy should be considered, and the use of triple‐therapy immunosuppression deserves further study.

### 3.4 Cases in the veterinary literature that have undergone ALK and PK—comments on outcome and graft rejection

A total of 176 canine eyes from patients with a variety of ophthalmic diseases, including deep corneal ulcers and perforations (*n* = 158), as well as limbal melanocytoma (*n* = 12),^50^, ^69^ pigmentary keratitis (*n* = 2),^42^, ^43^ corneoscleral thinning, stromal abscess, interstitial keratitis, and malignant iris melanoma with corneal involvement (*n* = 1 each)^34^, ^43^ that were treated with ALK or PK, were included in the veterinary literature. Surprisingly, only 8/158 (6%) of the eyes with corneal ulcers/perforations had preoperative results on aerobic and anaerobic bacterial culture with susceptibility testing (see the “Corneal transpositions” part of the “Autogenous ocular tissue” section of this review for more information).^35^

Despite the immune privilege and tolerogenic mechanisms associated with the anterior segment of the eye,^48^ graft rejection exists, with incidences ranging from 1.5% to 65%.^46^, ^47^ This significant discrepancy may be due to differences in what is considered as a graft rejection. The main discrepancy is associated with corneal vascularization: While some authors do not consider an increase of vascularization as a sign of rejection *per se*,^45^, ^47^ but a risk factor for rejection, others do.^46^ The rejection rate clearly depends on the immunologic/clinical state of the recipient cornea. Thus, canine keratoplasties for the treatment of noninflammatory corneal dystrophies could have higher success rates than keratoplasties for acquired inflammatory corneal diseases. Corneal inflammation, infection, and/or vascularization in the latter are responsible for most of the rejected or failed grafts. Unfortunately, nearly all the canine eyes with deep corneal ulcers or perforations show inflammation, infection, and/or vascularization (i.e., they are “hot eyes”) and carry a high risk of corneal rejection because of it.

Predicting visual outcomes and potential postoperative complications after keratoplasty is not an easy task. Outcomes may range from complete recovery of corneal integrity with perfect transparency to severe conditions warranting enucleation. In the dog, visual acuity recovery although considered a goal is not the main objective of reconstructive keratoplasties, as maintaining adequate spatial orientation of the animal may be in most cases considered a satisfactory outcome. Reported visual outcomes for the treatment of deep ulcers and corneal perforations in dogs are relatively high, varying between 69.5% and 82%.^46^, ^47^ Nevertheless, comparing those outcomes is also complex, as depend on many additional factors, such as age, breed and facial conformation, owner compliance when administering treatment, size, origin, cryopreservation method, location, and thickness of the graft, suture material and knotting, corneal infection and inflammation, other concurrent ocular and systemic diseases. In addition, the expertise of the surgeon in keratoplasties plays an important role in the long‐term success.^61^ The most common postoperative complications of keratoplasties are transplant rejection, suture dehiscence, and corneal opacities of various degrees.^45^, ^46^, ^47^

Despite the differences in grading systems, optically clear corneas with mild scarring have been obtained in 40.4% (23/57) and 27.5% (8/29) of the ulcerated/perforated eyes treated with cryopreserved heterografts.^46^, ^47^ It is worth highlighting that achieving complete transparency of the corneal cryopreserved grafts after PK surgery is challenging, as no viable endothelium is provided. Nevertheless, migration of endothelial cells adjacent to the donor button could allow for the frozen graft to become transparent months after the surgery, as has been described before for autologous grafts.^34^ In addition, brachycephalic breeds, and especially Pugs, are predisposed to pigmentary keratitis,^65^ and this poses a risk to grafted corneal tissues.^46^, ^70^

## 4 CORNEAL CLARITY

A lack of standardization on the assessment of postoperative graft opacification occurs when authors evaluate patients at different end time points and employ different grading systems. As shown in the present review, the central cornea is frequently affected by CUD, meaning that reconstructive corneal surgery commonly involves the visual axis. Efforts have to be made in order to encourage the standardization of the reporting of results for central corneal clarity in small animals undergoing corneal surgery, or that have corneal disease that affects central corneal clarity.^71^

**FIGURE 1:**
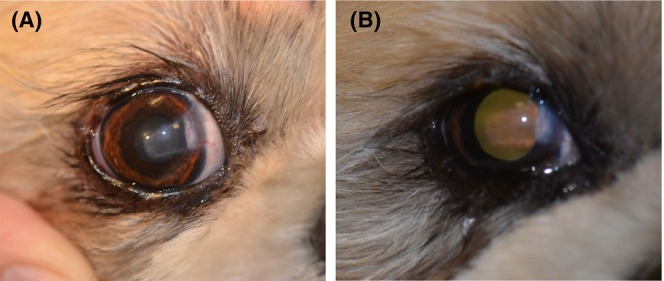
The eye in the images has undergone a CLCT graft several weeks prior to the pictures being taken by the same camera (Nikkon D7000 with a standard camera‐mounted flash) in the same day, seconds apart. One image (A) shows the eye lit up by the flash with the cornea in focus, while the second image (B) has been taken with the same camera and flash but showing the tapetal reflection and allowing the camera to automatically focus (i.e., in this instance, on the lateral iris). The differences in lighting show the same CLCT at nearly the same moment but looking very different. The first image (A) enhances the corneal scar, and the second image (B) shows how the tapetal reflection is able to cross the cornea unimpeded through most of the CLCT. Despite being taken only seconds apart, the images might be interpreted by the observer or an imaging processing system as showing different degrees of healing based on the apparent degree of corneal scarification (i.e., it is more obvious in image A than in image B). The use of both images, or one of the two should be encouraged in studies for all eyes when images are shown, but should not exclude the use of corneal clarity scoring.

Corneal images used in studies to demonstrate the presence of corneal clarity are subject to lighting conditions that change the appearance of the cornea and surrounding structures and can affect the impression given to the person judging the images (Figure 1). Ideally, corneal clarity would always be assessed clinically by the examiner through a scoring system that meets certain requirements: It avoids vague terminology (e.g., “moderate amount” of vascularization present, or “it is possible but difficult to see through” without detailing what specifically the observer is supposed to see); it contains clear descriptors for each score category (e.g., “the fine surface architecture of the iris is visible,” or “the fine details of the expected fundic anatomy for that species are clearly visible”); it contains categories with descriptors written in a clear and concise form; it has been developed for the purpose and species being used (i.e., central corneal scarring after disease and/or surgery in dogs and cats); and it has undergone inter‐ and intra‐user variability testing in clinical cases that fit the purpose and the species for which the scoring system is specifically designed for or adapted to.^71^

Forty‐two of the corneal surgery articles cited in this review were evaluated to determine the way central corneal clarity was reported by the authors. A total of 14 studies were excluded because the disease process did not directly affect the central cornea (i.e., limbal melanocytic masses), tissues were used that were not expected or likely to result in transparency although central corneal defects were present (i.e., grafts made with tarsoconjunctiva, buccal mucosal grafts, grafts of conjunctiva, renal capsule, or pericardium), or the data on corneal clarity were not found, which left a total of 28 studies. These were divided into five categories (i.e., C1‐C5): (C1) No scoring system was used but observational comments were included; (C2) No scoring system was used though at least one specific descriptor (i.e., “being able to see the fundus”) was included, or the authors used a scoring system (i.e., mild, moderate, marked, and severe) that contained no specific descriptors for each category; (C3) a scoring system with specific descriptors was used but it included vague terminology (e.g., “mild vs. moderate amounts” of vascularization present); (C4) a scoring system with specific descriptors was used and it contained no vague terminology, but the scoring system had not undergone an inter‐user/intra‐user variability analysis for the species/purpose for which it was being used (i.e., central corneal clarity of small animal patients); and (C5) a scoring system with specific descriptors was used that contained no vague terminology and that had undergone an inter‐user/intra‐user variability analysis for the species/purpose it was being used for. There were a total of 15/28 (53.57%) articles in category C1, 6/28 (21.43%) articles in category C2, 3/28 (10.71%) articles in category C3, 2/28 (7.14%) articles in category C4, and 2/28 (7.14%) articles in category C5. These results highlight there were significant differences between studies based on the scoring system used. This could be minimized if clinicians adopted scores that fit, at least, category C4 and, ideally, category C5.

## 5 CONCLUDING COMMENTS

Animal, disease, and surgical factors impacting graft survival and ultimate corneal clarity following corneal reconstructive surgery remain poorly understood in veterinary ophthalmology.

The majority of the dogs in this review were affected with CUD with the rest affected by degenerative corneal conditions or limbal masses. Corneal ulcerative disease frequently affected brachycephalic dogs and a large number of affected eyes presented as perforations or near perforations (i.e., descemetoceles), suggesting CUD is recognized late, referred late, and/or it developed rapidly in those cases, while the proportion of cases that could not be referred or that healed and did not require referral is not known. When described, CUD often occurred centrally, which is expected to affect vision. Grossly, similar surgical success rates for different techniques were reported across studies, though corneal transplant studies showed most variation in results. Most studies focused on single techniques and only a few studies investigated possible differences between variations of a particular technique.

Inconsistent study design and data reporting make direct comparison between publications and graft types challenging. A variety of limiting factors were recognized in many of the veterinary corneal reconstruction reports included in this review, which allowed for the creation of general recommendations to help minimize some of the challenges encountered by surgeons when comparing studies.

The most important limitations are summarized here. Studies with 50 patients or more were rare, and with 100 patients or more were scarce. Patient signalment was occasionally omitted, and the history of previous CUD was rarely reported (i.e., whether it was historical evidence or based on findings of the ophthalmic examination) and authors often did not mention if culture and sensitivity testing was carried out. Other aspects varied considerably, such as the definition of success. It would seem logical this would include if tectonic support was achieved and, when applicable, if it maintained a sealed the anterior chamber, if the flap/graft remained integrated into the cornea, and how corneal clarity scoring was carried out. The follow‐up times varied widely between studies as well. Despite variations in appointment and owner availability, there are times at which it is of interest to see a patient, including, but not exclusively at the time of removal of the protective collar, when absorbable sutures are expected to dissolve, and when most of the cornea has cleared. In the authors' experience, these tend to occur around 7–14 days, 6–8 weeks, and 3–4 months postoperatively, respectively, though studies need to identify these times more accurately. Lastly, the reporting of clarity scoring was often subjectively and insufficiently assessed.

Based on these limiting factors, several recommendations are made. Reports of large groups of animals should be encouraged. Patient signalment, history of previous CUD, if culture and sensitivity were carried out, and all key aspects of the surgical technique used should always be reported. Definitions of success should include comments on tectonic support, anterior chamber stability, graft integrity, and comments on transparency. Clinicians should plan re‐evaluations at key times throughout the course of the healing process when significant changes are expected to occur and report corneal clarity scoring at each of those times using a corneal clarity scoring system that minimizes inaccuracies through objective means and a design tailored for use in a standard referral clinical setting. Lastly, studies that are designed and executed to compare different corneal reconstructive techniques, and studies that improve our understanding of how the recipient endothelium might interact with donor tissue of grafts that do not contain endothelium, should be encouraged.

## 6 ETHICS STATEMENT

This study complies with the Guidelines for Ethical Research in Veterinary Ophthalmology (GERVO) and is exempt from approval by an ethics committee. The client owned patient images were taken with written permission of the client.

## Data Availability

Data sharing not applicable to this article as no datasets were generated or analyzed during the current study. All the reviewed articles are part of the veterinary and medical literature.
